# Congenital Upper Eyelid Coloboma: Clinical and Surgical Management

**DOI:** 10.1155/2015/286782

**Published:** 2015-08-23

**Authors:** José María Ortega Molina, Eduardo Ramón Mora Horna, Andrés David Salgado Miranda, Rosa Rubio, Ana Solans Pérez de Larraya, Guillermo Salcedo Casillas

**Affiliations:** ^1^Department of Ophthalmology, San Cecilio University Hospital, Avenida Dr. Olóriz 16, 18012 Granada, Spain; ^2^Orbital and Oculoplastic Service, Asociación para Evitar la Ceguera en México I.A.P. “Dr. Luis Sánchez Bulnes”, Mexico City, DF, Mexico

## Abstract

*Purpose*. The goal was to describe our experience in the surgical management and treatment of four patients with congenital upper eyelid colobomas. *Methods*. A descriptive, observational, retrospective study was performed including patients with congenital eyelid colobomas referred to Asociación para Evitar la Ceguera en México I.A.P. “Dr. Luis Sánchez Bulnes” between 2004 and 2014 and assessed by the Oculoplastics and Orbit Service. *Results*. The four cases required surgical treatment of the eyelid defects before one year of age and their evolution was monitored from the time of referral to the present day. One of the patients needed a second surgical procedure to repair the eyelid defect and correct the strabismus. *Conclusions*. Eyelid colobomas are a potential threat to vision at an early age, which requires close monitoring of the visual development of patients.

## 1. Introduction

Congenital eyelid coloboma is an uncommon, unilateral or bilateral, partial or full-thickness eyelid defect. It is caused by failure of fusion of the mesodermal lid folds [1–3].

It may be isolated or associated with other ocular or systemic anomalies. Immediate attention at an early age through corneal protection, surgical repair of the eyelid defect, and monitoring of the visual development are essential to prevent complications: corneal leukoma, symblepharon, and amblyopia [4–6].

This report summarizes our experience in the surgical management and treatment of four patients with congenital eyelid colobomas.

## 2. Methods

A descriptive, observational, retrospective study was performed including patients with congenital eyelid coloboma referred to Asociación para Evitar la Ceguera en México I.A.P. “Dr. Luis Sánchez Bulnes” between 2004 and 2014 and assessed by the Oculoplastics and Orbit Service.

A detailed clinical history was collected, including information about personal and family history, exposure to drugs or diseases during pregnancy, and a complete ophthalmic examination at the time of referral, at subsequent check-ups, and in the postoperative period.

An initial examination was performed to assess the visual acuity (VA), site, and size of the eyelid defect and ocular motility as well as to determine the presence or absence of ocular anomalies (by biomicroscopic examination, examination of the ocular fundus, and ocular ultrasonography) and other facial or systemic anomalies.

## 3. Results

This study included four patients with eyelid coloboma associated with other ocular and systemic pathologies (Table 1).

The incidence of unilateral eyelid involvement (two cases) was found to be the same as that of bilateral eyelid involvement (two cases). The most frequently associated ocular anomaly was dermoid cyst (two cases) and symblepharon (two cases). One of the patients presented with strabismus; therefore, surgery was performed. None of them had glaucoma, cataract, iris, retinal, or optic nerve coloboma. The mothers of the patients had no history of infections or other illnesses during pregnancy and there was no exposure to drugs or medicines.

## 4. Case Reports

### 4.1. Case  1

**Figure 1:**
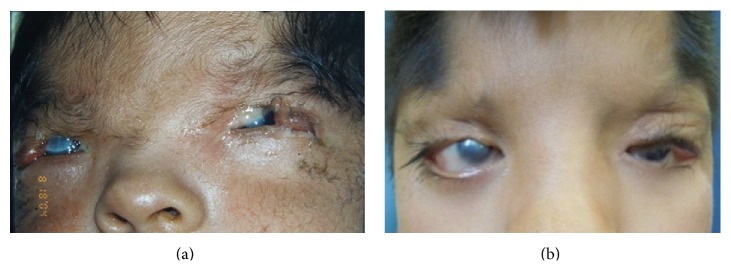
(a) Case  1 with 17 days old. The patient has bilateral eyelid coloboma and bilateral corneal erosions due to exposure. (b) Case  1. At the age of 9 years after surgery.

A 17-day-old male infant was referred to us for congenital upper eyelid coloboma of medial location involving more than two-thirds of the eyelid margin. The patient had telecanthus, aberrant anterior hairline, partial absence of eyebrows, and hypoplasia of nasal bridge. Ocular examination revealed the presence of corneal erosion involving more than 80% of the corneal surface in both eyes (OU), conjunctival dermoid cyst, and symblepharon in the left eye (OS). Ultrasonography of both eyes was normal and showed orthotropia (Figure 1(a)). Resection of dermoid cyst and reconstruction of OU using Tenzel's semicircular flap plus canthotomy and cantholysis were performed.

Currently, after eight years of surgery, the patient has 0.5 mm of lagophthalmos in the OD, 1 mm in the left eye, and 20/200 of VA due to the presence of corneal leukomas, remaining stable and without symptoms (Figure 1(b)).

### 4.2. Case  2

A 9-year-old female patient was referred due to congenital upper eyelid coloboma in the OD affecting the medial one-half of the upper eyelid. She had undergone surgery in the first month of life for correction of the defect. The patient had more than 4 mm of lagophthalmos, euryblepharon, superior temporal symblepharon, central corneal leukoma, and 30-degree esotropia. Ocular ultrasonography of the OD was normal.

Joint surgery was performed to correct esotropia and close the eyelid defect. In order to correct esotropia, lateral rectus recession and medial rectus recession were performed. The eyelid defect was repaired with a Mustarde rotational flap which was opened 4 weeks later.

Despite the initial success of the surgery, the patient had an incomplete eyelid closure; therefore, therapeutic contact lenses and ocular lubricants were applied, remaining stable and without symptoms. The VA was 20/400 due to central corneal leukoma.

### 4.3. Case  3

A 1-month-old male infant was referred to our service due to upper eyelid coloboma in the OS involving the medial one-third of the upper eyelid. He had a bifid nose, ogival palate, abnormal palmar creases, and imperforate anus. The patient had orthotropia and the ocular ultrasonography was normal. Tenzel's semicircular flap was performed. Currently, the patient is 3 years old, has a VA of 20/20, and has no other ophthalmological alterations.

### 4.4. Case  4

A 5-month-old male infant was referred to our service due to upper bilateral eyelid coloboma. In the OD, it involved the medial one-third of the upper eyelid and in the OS, there was a small notch less than 3 mm.

He presented with three bilateral preauricular appendages and one dermoid cyst in the OS. The patient had orthotropia and the ocular ultrasonography in OU was normal. In this case, based on the phenotype and anomalies of the patient, the Genetics Service made the diagnosis of Goldenhar syndrome. Tenzel's semicircular flap combined with a canthotomy and cantholysis of the upper eyelid was performed.

**Figure 2:**
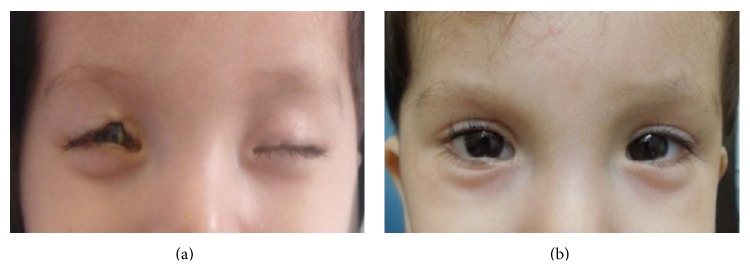
(a) Case  4 with 5 months old. The patient has bilateral eyelid coloboma. (b) Case  4. At the age of 2 years after surgery.

Currently, after four years of surgery, the patient has a VA of 20/20, has complete eyelid closure, and has no other anomalies (Figures 2(a) and 2(b)).

## 5. Discussion

There are several theories that attempt to explain the cause of congenital upper eyelid colobomas. Tessier considers coloboma of the eyelid to be a form of facial cleft [7]. Other authors suggest that these colobomas are associated with intrauterine factors such as amniotic bands, abnormal fetoplacental circulation, or radiation [4, 8, 9].

They are usually unilateral, generally located at the medial one-third of the upper eyelids (90%), and may vary from a small notch to complete defects of the eyelid.

Lower eyelid colobomas usually affect the outer one-third of the eyelid [5, 10, 11].

Colobomas are also associated with facial clefts, Goldenhar syndrome, Treacher Collins syndrome, Charge syndrome, or frontonasal dysplasia [5, 6, 12].

They may present other ocular and orbital anomalies such as conjunctival or limbal dermoid tumors, conjunctival chondroma, symblepharon, corneal opacities, macular or optic nerve colobomas, and strabismus [9, 13].

Eyelid reconstruction at the right time is essential in these patients. This will depend on the size of the defect and on the presence of corneal exposure.

If the defect is small and there is no corneal exposure, surgery could be delayed until the age of 3-4, when there is an increased amount of eyelid tissue. Otherwise, surgery should be done as soon as possible to avoid corneal lesions [5, 6, 14, 15].

The surgical technique will depend on the size of the defect. Defects up to 25% can be closed directly. Defects between 25% and 50% can be repaired by direct closure with canthotomy and cantholysis or by Tenzel's semicircular flap. In case of defects greater than 50% of the eyelid, functional and cosmetic results will be difficult to achieve. In these cases, several techniques could be used such as Tenzel's semicircular flap, Cutler-Beard technique, or Mustarde rotational flap [2, 3, 5, 6, 15].

Other techniques have been described such as the lamellar-based technique, proposed in patients with a discordant defect between the anterior and the posterior lamellae of the eyelid [11]. Others such as tarsomarginal grafts allow the defects involving more than 50% of the lid margin to be closed and all eyelid structures to be replaced [5].

## 6. Conclusions

Congenital upper eyelid coloboma is an uncommon defect of unknown etiology, which might be associated with other ocular and systemic pathologies, and therefore it requires a multidisciplinary approach in a large number of cases.

Eyelid colobomas are a potential threat to vision at an early age, requiring close monitoring of the visual development of patients.

The surgical technique and timing of surgery depend on the size of the defect and the presence or absence of corneal exposure.

In case of eyelid colobomas involving more than 50% of the lid margin, both cosmetic and functional surgical results are difficult to obtain. In such cases, provided that there is no media opacity, one-stage techniques will be preferable since they do not involve the visual axis and therefore, there is no risk of amblyopia.

## Figures and Tables

**Table 1:** Summary of cases.

Sex	Age	Ocular manifestations	Systemic manifestations	Surgical treatment
Male	17 days	Bilateral upper eyelid coloboma involving the medial two-thirds of the upper eyelid Telecanthus Conjunctival dermoid cyst Symblepharon	Nasal hypoplasia	Tenzel's semicircular flap Canthotomy + cantholysis

Female	9 years	Unilateral upper eyelid coloboma involving the medial one-half of the right eye Symblepharon Corneal leukoma Esotropia Euryblepharon Lagophthalmos	Madarosis Aberrant anterior hairline Absent right eyebrow	Lateral Rectus resection + medial rectus recession Mustarde rotational flap

Male	1 month	Unilateral upper eyelid coloboma involving the medial one-third of the left eye	Bifid nose Ogival palate Abnormal palmar creases Imperforate anus	Tenzel's semicircular flap

Male	5 months	Bilateral upper eyelid coloboma involving the medial one-third of the right eye and small notch in the left eye Limbal dermoid cyst in the left eye	Goldenhar syndrome Bilateral preauricular appendages	Tenzel's semicircular flap Canthotomy + cantholysis
